# Use of topical glutamine as an adjuvant for the treatment of oral ulcers

**DOI:** 10.5935/0103-507X.20180047

**Published:** 2018

**Authors:** Aline Bergman de Souza Herculano, Ellen Cristina Gaetti-Jardim, Débora Pereira de Aurélio, Guilherme Soares da Silva, Janayna Gomes Paiva Oliveira, Gustavo Silva Pelissaro, José Carlos Garcia de Mendonça

**Affiliations:** Universidade Federal de Mato Grosso do Sul - Campo Grande (MS), Brasil.; Hospital Universitário Maria Aparecida Pedrossian - Campo Grande (MS), Brasil.; Faculdade de Odontologia, Universidade Federal de Mato Grosso do Sul - Campo Grande (MS), Brasil.

**To the Editor**

Glutamine is the most commonly found free amino acid in the human body. When applied to clinical lesions, glutamine can aid in the recovery of severe presentations, reduce infections and even decrease the length of hospital stay because it causes symptom remission. For patients with severe systemic presentations, this amino acid is classified as essential because it is required by the body. Glutamine can reduce the inflammatory response, antagonize prostaglandins and regulate the activities of cytotoxic natural killer (NK) lymphocytes^(1)^ and neutrophils;^(2)^ reduction of its serum level is extremely harmful to the body.

A 35-year-old male patient was admitted to the Intensive Care Center of the Maria Aparecida Pedrossian University Hospital, in Campo Grande, Mato Grosso do Sul (MS), Brazil, with complications of HIV. After examination, he was referred to the Infectious and Parasitic Diseases Sector, where he was treated for lymph node tuberculosis. Due to clinical manifestations caused by orotracheal tube use and worsening HIV symptoms, perioral and oral lesions with spontaneous bleeding were present on all injured extremities (Figure 1A and B), making it impossible for the patient to speak or swallow, in addition to causing pain. As an adjuvant treatment, topical glutamine was applied over the affected area, which was combined with dietary glutamine supplementation. Application was performed in the morning after aspiration of oropharyngeal contents and oral hygiene with 0.12% chlorhexidine solution. The amount used for topical application was sufficient to cover the entire lesion (Figure 1C).

**Figure 1:**
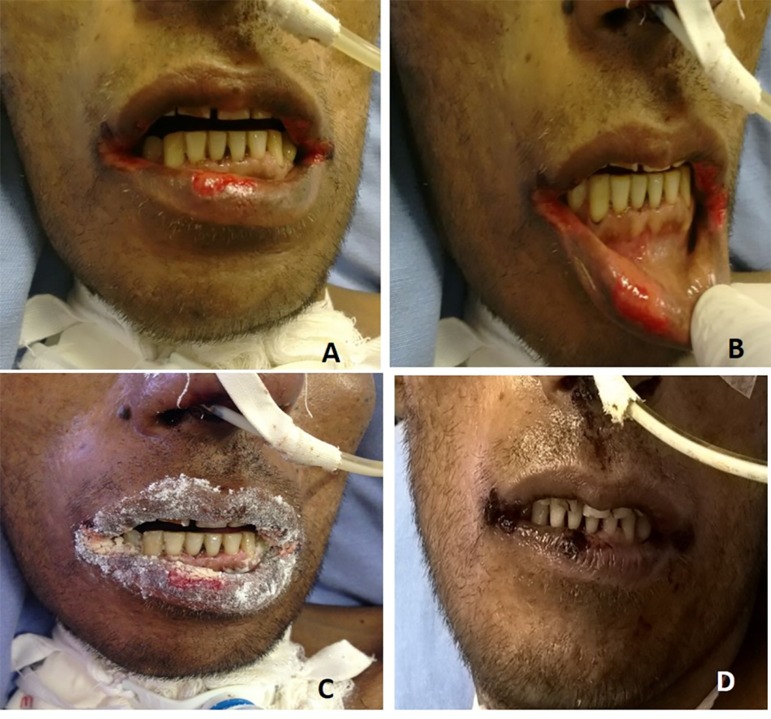
Clinical aspect before and after treatment with glutamine (A and B). Multiple lesions on the lips and mouth corners (C). Glutamine application on the lip and mouth corner lesions. (D) Improved lesion appearance and presence of a scab.

Treatment continued for 1 week. On the second day of application, bleeding was absent, and scab formation was observed on the lower lip and both mouth corners, giving the appearance of a decrease in lesion size, which was evident 7 days later (Figure 1D). The most relevant point was the reduction of the patient's symptomatology because the lesions to which the topical amino acid was applied showed regression and significant improvement during the subsequent applications, resulting in a higher quality of life for the patient. After improvement of the systemic condition, the patient was discharged and directed to perform home treatment.

The appearance of opportunistic lesions, especially ulcers, in immunosuppressed patients may be an important clinical indication of their immunological status. In this sense, glutamine can act as adjuvant treatment because its use has been described in the literature for oral mucositis in cancer patients, with promising results.^(3,4)^

HIV patients often present with oral manifestations such as candidiasis, gingivitis, volumetric increases such as papillomas and ulcerations, which can cause great discomfort, pain, altered taste and, consequently, loss of quality of life.^(5)^ In the reported case, the application of topical glutamine helped protect the ulcerated areas in the labial mucosa, reducing the patient's pain and facilitating feeding. An increase in the epithelization of the affected region occurred due to reduced saliva accumulation at the site and a lack of exposure to the environment.

*Aline Bergman de Souza Herculano* *Universidade Federal de Mato Grosso do Sul - Campo Grande (MS),
Brazil.* *Ellen Cristina Gaetti-Jardim* *Universidade Federal de Mato Grosso do Sul - Campo Grande (MS),
Brazil.* *Débora Pereira de Aurelio* *Hospital Universitário Maria Aparecida Pedrossian - Campo Grande
(MS), Brazil.* *Guilherme Soares da Silva* *Faculdade de Odontologia, Universidade Federal de Mato Grosso do Sul - Campo
Grande (MS), Brazil.* *Janayna Gomes Paiva Oliveira* *Hospital Universitário Maria Aparecida Pedrossian - Campo Grande
(MS), Brazil.* *Gustavo Silva Pelissaro* *Hospital Universitário Maria Aparecida Pedrossian - Campo Grande
(MS), Brazil.* *José Carlos Garcia de Mendonça* *Faculdade de Odontologia, Universidade Federal de Mato Grosso do Sul - Campo
Grande (MS), Brazil.*

## References

[1] Klimberg VS, Kornbluth J, Cao Y, Dang A, Blossom S, Schaeffer RF (1996). Glutamine suppresses PGE2 synthesis and breast cancer
growth. J Surg Res.

[2] Lagranha CJ, Levada-Pires AC, Sellitti DF, Procopio J, Curi R, Pithon-Curi TC (2008). The effect of glutamine supplementation and physical exercise on
neutrophil function. Amino Acids.

[3] Chattopadhyay S, Saha A, Azam M, Mukherjee A, Sur PK (2014). Role of oral glutamine in alleviation and prevention of
radiation-induced oral mucositis: A prospective randomized
study. South Asian J Cancer.

[4] Miranda MP, Souza DS (2015). Glutamina na prevenção e tratamento da mucosite em
pacientes adultos oncológicos: uma revisão sistemática
da literatura. Rev Bras Cancerol.

[5] Donoso-Hofer F (2016). Lesiones orales asociadas con la enfermedad del virus de
inmunodeficiencia humana en pacientes adultos, una perspectiva
clínica. Rev Chilena Infectol.

